# Aberrant expression of bone morphogenetic proteins in the disease progression and metastasis of breast cancer

**DOI:** 10.3389/fonc.2023.1166955

**Published:** 2023-06-02

**Authors:** Ming Liu, Laijian Sui, Ziqian Fang, Wen G. Jiang, Lin Ye

**Affiliations:** ^1^ Cardiff China Medical Research Collaborative, Division of Cancer and Genetics, Cardiff University School of Medicine, Cardiff, United Kingdom; ^2^ Department of Surgery, Shandong University of Traditional Chinese Medicine Affiliated Hospital, Jinan, Shandong, China

**Keywords:** BMP, breast cancer, metastasis, progress, subtype

## Abstract

**Background:**

Bone morphogenetic proteins (BMPs) play crucial roles in the tumorigenesis and metastasis of cancers. Controversy remains about the exact implications of BMPs and their antagonists in breast cancer (BC), due to their diverse and complex biological functions and signalling. A comprehensive study of the whole family and their signalling in breast cancer is provoked.

**Methods:**

Aberrant expression of BMP, BMP receptors and antagonists in primary tumours in breast cancer were analysed by using TCGA-BRCA and E-MTAB-6703 cohorts. Related biomarkers including ER, HER, proliferation, invasion, angiogenesis, lymphangiogenesis and bone metastasis were involved to identify the relationship with BMPs in breast cancer.

**Results:**

The present study showed BMP8B was significantly increased in breast tumours, while BMP6 and ACVRL1 were decreased in breast cancer tissues. The expressions of BMP2, BMP6, TGFBR1 and GREM1 were significantly correlated with BC patients’ poor overall survival. Aberrant expression of BMPs, together with BMP receptors, were explored in different subtypes of breast cancer according to ER, PR and HER2 status. Furthermore, higher levels of BMP2, BMP6 and GDF5 were revealed in triple negative breast cancer (TNBC) whilst BMP4, GDF15, ACVR1B, ACVR2B and BMPR1B were relatively higher in Luminal type BC. ACVR1B and BMPR1B were positively correlated with ERα but were inversely correlated with ERβ. High expression of GDF15, BMP4 and ACVR1B were associated with poorer overall survival in HER2 positive BC. BMPs also play dual roles in tumour growth and metastasis of BC.

**Conclusion:**

A shift pattern of BMPs was showed in different subtypes of breast cancer suggesting a subtype specific involvement. It provokes more research to shed light on the exact role of these BMPs and receptors in the disease progression and distant metastasis through a regulation of proliferation, invasion and EMT.

## 1 Introduction

Breast cancer has overtaken lung cancer, becoming the leading cancer, although lung cancer remains the most lethal cancer, with the highest mortality in 2020 (1). Breast cancer, at early stage and small size tumours without metastasis, is related to better patients’ survival, lower incidence and reduced mortality. Patients with early stage breast cancer may benefit from treatments including surgery and regional radiotherapy, chemotherapy, endocrine therapy, anti-HER2 therapy, and also immunotherapy and cell-based therapies, developed in recent years (2). However, understanding and treatment of metastatic BC remain poor.

BMPs are members of the TGF β (transforming growth factor β) superfamily. Previous studies have reported that there are over 20 BMP ligands, which are transduced by seven type I and five type II transmembrane serine/threonine kinase receptors (3). They were originally found in bones, playing a critical role in endochondral bone growth, embryonic development, tissue repair and homeostasis and stemness (4). Upon interaction with the BMP ligands, the Type-II receptors phosphorylate the Type-I receptors. This leads to recruitment of the pathway-restricted Smads (R-Smads, Smads1, 5 and 8) to the receptor complex. The intercellular signalling complexes of R-Smads then translocate into the nucleus. This pathway is known as the Smad-dependent pathway (5). The other pathway, known as the Smad independent pathway, does not require Smads, but involves the MAP kinase pathways (P38, JNK and ERK), PI3K/AKT, nuclear factor κβ (NF‐κβ) and Ras homolog (Rho), etc (6).

Overexpression of BMP2 has been associated with poor disease-free survival by enhancing AKT/mTOR pathway among 272 patients with breast cancer (7). Previous research demonstrated that BMPs, as well as TGF-β, were able to promote invasion and bone metastasis in breast cancer *in vivo*. BMP2 and TGF-β3 enhanced motility of MDA-MB-231 cells *in vitro* as well as in the xenograft model (8). From a study by Buijs and colleagues, decreased expression of BMP7 in primary tumours is significantly related to bone metastasis in breast cancer. BMP7 can inhibit cell proliferation and differentiation of breast cancer cells at primary sites and also in bone (9). Additional evidence for the involvement of BMPs in bone metastasis came from another observation that BMP2/BMP7 heterodimer inhibited colonisation of breast cancer cells in bone (10). BMP6 increased E-cadherin and promoted cell adhesion and invasion by repressing δEF1 at mRNA level in MCF-7 and MDA-MB-231 cells (11).

To date, the role played by BMPs in breast cancer remains controversial and needs to be further investigated and dissected. In the current research, we aim to evaluate the diverse role of BMP ligands and receptors in breast cancer by analysing publicly available datasets of breast cancer.

## 2 Methods and datasets

### 2.1 Breast cancer datasets

#### 2.1.1 Breast cancer cohort from the TCGA-BRCA

The data was derived from breast cancer samples in The Cancer Genome Atlas (TCGA) (https://portal.gdc.cancer.gov) database. RNA sequencing dataset comprises 1098 primary breast tumours, 112 adjacent normal tissues and 6 metastatic tumours. The expression of BMPs, BMP receptors and antagonists in breast cancer tissues (n=1093) were analysed in comparison with adjacent normal tissues (n=112). This cohort was employed as a dataset for analysis of correlation between BMPs and markers including ER, HER2, proliferation markers (KI67 and PCNA), EMTs (SNAI, TWIST, VIM, ZEB, CDH2), MMPs, angiogenesis (CD34, VEGFR), lymph angiogenesis (PDPL, LYVE) and bone metastasis.

#### 2.1.2 Breast cancer cohort from the Gene Expression Omnibus database

GEO is an open gene expression repository including microarray data, sequencing data analysis and other genomics data.

GSE70951. A gene expression database (12) contains tumour samples of breast adenocarcinoma (n=47), paired adjacent normal breast tissues (n=47) and normal breast tissues derived from mammaplastic reduction (n=43).

GSE27473. Influence of oestrogen receptor (ER) on the expression of BMPs was evaluated in GSE27473 (13) dataset, which comprises MCF-7 derived ER silencing samples (n=3) and controls (n=3).

E-MTAB-6703. A meta-dataset comprises of 2302 samples including both primary tumours and tumour-free breast tissues from 20 independent GEO datasets. Correlated genes, identified from the discovery cohort (TCGA_BRCA) were further validated in this dataset.

E-MTAB-4003. This gene array dataset comprises primary tumours from patients with distant metastasis to bone only (n=127), visceral organs (n=167), bone and visceral organs (n=173) and without distant metastasis (n=226).

### 2.2 Statistical analysis

T tests were used for normally distributed data. Mann-Whitney U tests were used to compare non-normally distributed data. For comparisons of multiple groups of data with normally distributed one-way ANOVA test was employed whilst the Kruskal-Wallis H test was used for non-normally distributed data. Survival analyses were performed using Kaplan-Meier survival analysis (KMplot) (https://kmplot.com) (14). Correlations between BMPs and different markers (proliferation, cell cycles, EMTs, MMPs) were evaluated using correlation test. Pearson’s correlation test for normally distributed data and Spearman’s correlation for non-normally distributed data were used. All data analyses were conducted using SPSS (IBM SPSS Statistics version 27, New York, USA). Heatmaps and scatter plots were plotted using GraphPad (GraphPad Prism 9, IBM Ltd, MA, USA). P<0.05 was considered as statistically significant.

## 3 Results

### 3.1 Aberrant expression and clinical implication of BMP and BMP signalling in breast cancer

**Figure 1:**
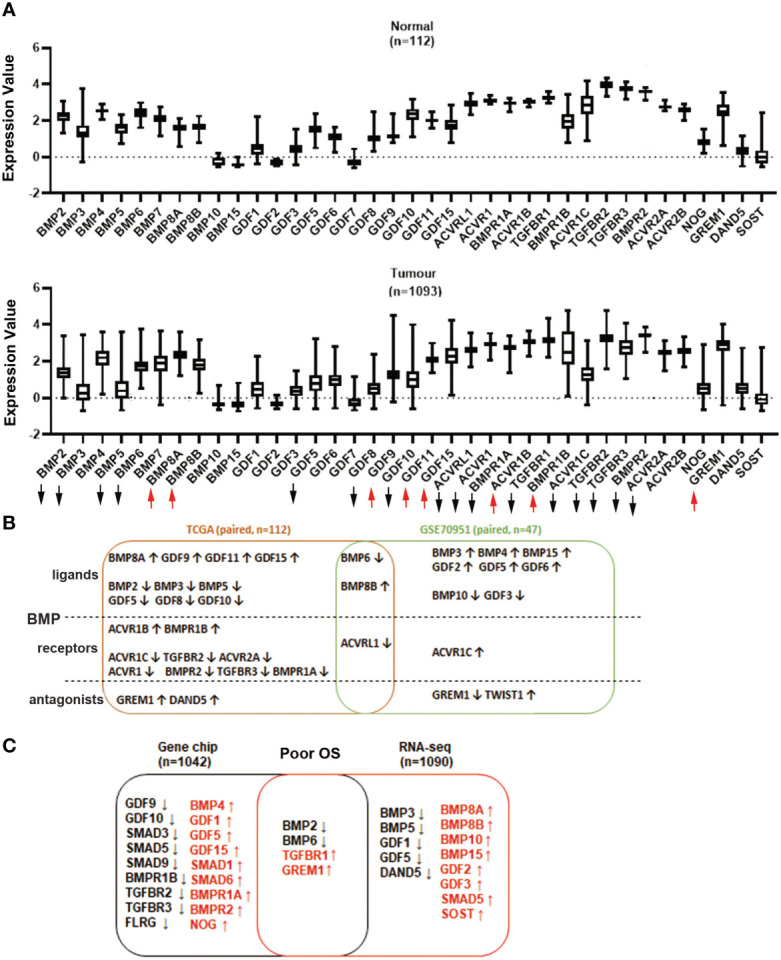
Aberrant expression of BMP ligands, BMP receptors and BMP antagonists in breast cancer. **(A)** Expression of BMPs in breast normal tissues (n=112) and tumour tissues (n=1093) were analysed using the TCGA dataset. **(B)** Shown are the overlapping BMPs, BMPRs and antagonists that were all expressed in both TCGA (n=112 paired) and GSE70951 (n=47 paired) cohorts. **(C)** Aberrant expression of BMPs, BMP receptors and BMP antagonists with significant overall survival in Gene chips (n=1042 overall survival patients) and RNA sequencing data (n=1090) of breast cancer cohorts which are derived from survival analysis conducted at the www.KMplot.com. Upward and downward arrows indicate a significant p value being less than 0.05. More details are available in the **Supplementary Tables 1-4**.

Expressions of BMP ligands, receptors and antagonists in breast cancer were first analysed using the TCGA breast cancer cohort. Shifts in expression patterns between those of normal tissues and tumour tissues were observed. The expressions of BMP2, BMP3, BMP5, BMP6, GDF5, GDF8, GDF10, ACVRL1, ACVR1, BMPR1A, TGFBR1, ACVR1C, ACVR2A, TGFBR2, TGFBR3 and BMPR2 were significantly decreased, whilst BMP8A, BMP8B, GDF9, GDF11, GDF15, ACVR1B, BMPR1B and GREM1 were significantly increased in breast tumours of the TCGA cohort (**Figure 1A**, **Supplementary Table 1**). Overlapping BMP6 and BMP8B expression were also evident in the GSE70951 cohort, which includes 47 paired tumours and normal breast tissues (**Supplementary Tables 2.1**-**2.3**). The expression of BMP receptors was also investigated in these 2 datasets. ACVR1C, TGFBR2, TGFBR3, ACVR2A, ACVR1, BMPR1A and BMPR2 were lower in breast tumours while ACVR1B and BMPR1B exhibited relatively higher expression levels in paired tumours compared with normal breast tissues, in the TCGA_BRCA cohort. Reduced levels of ACVRL1 were seen in both cohorts of breast cancer (**Figure 1B**).

Lower expression of BMP2 and BMP6, in the primary tumours, was correlated with poor overall survival (OS) in the RNA sequencing analysis (n=1090, **Supplementary Tables 3.1**-**3.4**). However, higher expression of BMP10 and BMP15 indicated poor OS. Decreased expression of BMP2, BMP6 and increased expression level of TGFBR1 and GREM1, were also evident in the Gene Chip dataset from the KMplot database (n=1042) (**Figure 1C**, **Supplementary Tables 4.1**-**4.4**).

We further evaluated the specific gene expression value in survival analysis by using the KMplot. Relatively lower expression of BMP2 and BMP6 were significantly associated with poor overall survival (OS) and relapse-free survival (RFS) (P<0.01). No obvious change was seen in the expression of BMP2 and BMP6 when the primary tumours developed distant metastases. On the other hand, high expression of TGFBR1 and GREM1 were significantly correlated with poor OS and distant metastasis free survival (DMFS), whilst the elevated GREM1 transcript levels are also associated with poor RFS (**Supplementary Figures S1A-C**).

### 3.2 Correlation between BMPs and oestrogen receptors

**Figure 2:**
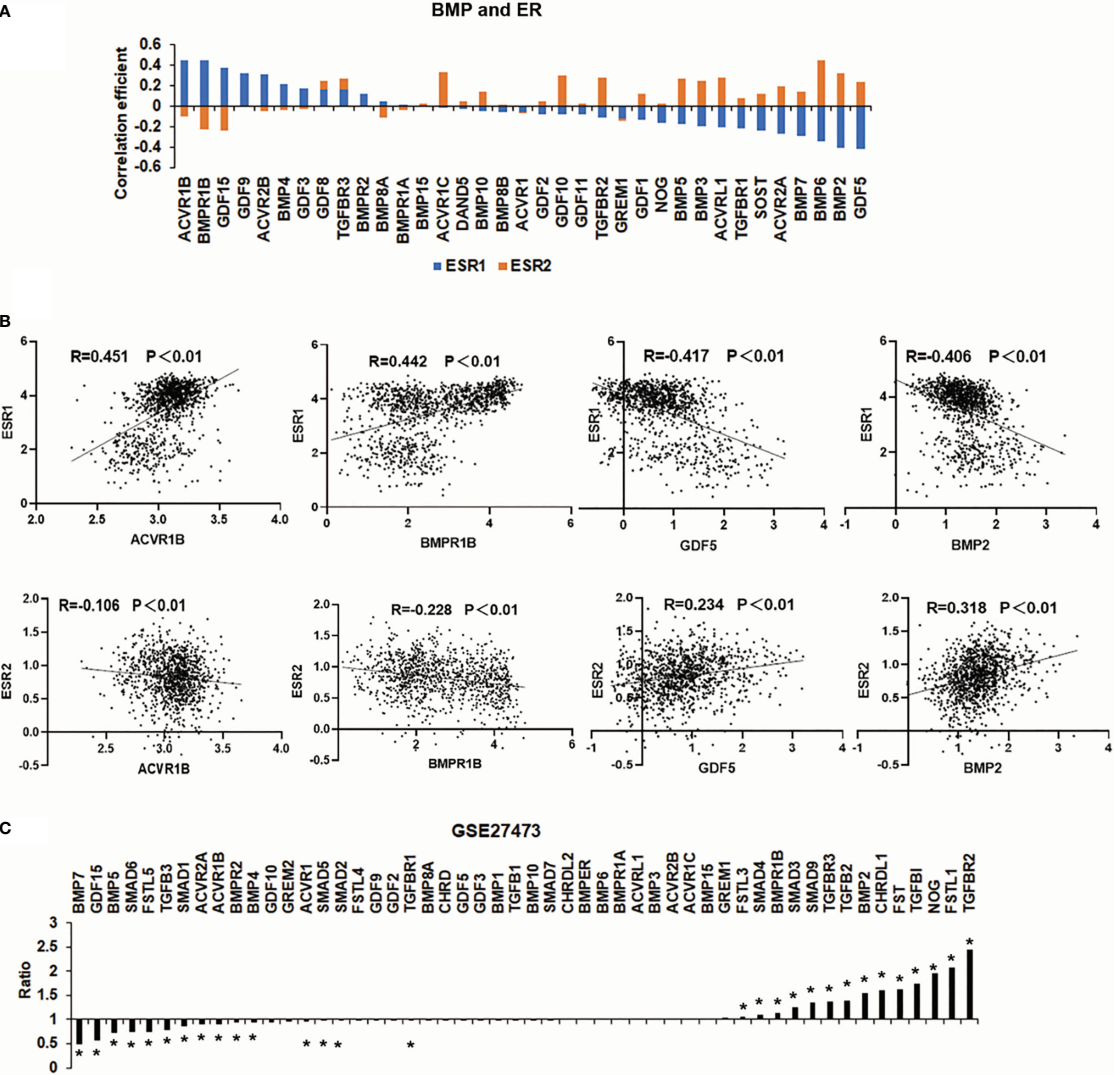
Correlation between BMPs and ER status. **(A)** The histogram shows the BMPs, BMPRs and antagonists are associated with ESR1 and ESR2 (ERα and ERβ). **(B)** Representative BMPs which were most correlated with ER are shown as scatter plots. **(C)** Aberrant expression of BMPs in MCF-7 cell line (GSE27473) of breast cancer are shown as a histogram plot. *Represents P<0.05.

ER, also known as ERα, is coded by the ESR1 gene. It is the most commonly used marker to characterise breast cancer. In addition to ERα, aberrant expression and function are also evident in breast cancer in relation to another oestrogen receptor ERβ, which is encoded by ESR2 (15). In the TCGA breast cancer cohort, most of the BMPs were significantly correlated with ESR1 (ERα) and ESR2 (ERβ) with a contrasting pattern. Interestingly, those BMPs positively correlated with ERα are more likely inversely correlated with ERβ (**Figure 2A**). For instance, the expression of ACVR1B, BMPR1B, BMP2 and GDF5 are more significantly correlated with ER. ACVR1B and BMPR1B are positively correlated with oestrogen receptor α transcripts (ERα) but are inversely correlated with oestrogen receptor β transcripts (ERβ). On the other hand, the correlation between BMP2, GDF5 and ESR1/2 were shown conversely (**Figure 2B**). To explore the relationship between BMPs and ER status, a dataset (GSE27473) which determined gene expression in MCF7 with ER silence was analysed. BMP7, GDF15, BMP5, SMAD6 and FSTL5 were highly expressed in the ER positive breast cancer cells (MCF-7 cells), while the expressions of TGFBR2, FSTL1 and NOG were reduced in this cohort (**Figure 2C**).

### 3.3 Expression of BMPs and HER family members in breast cancer

**Figure 3:**
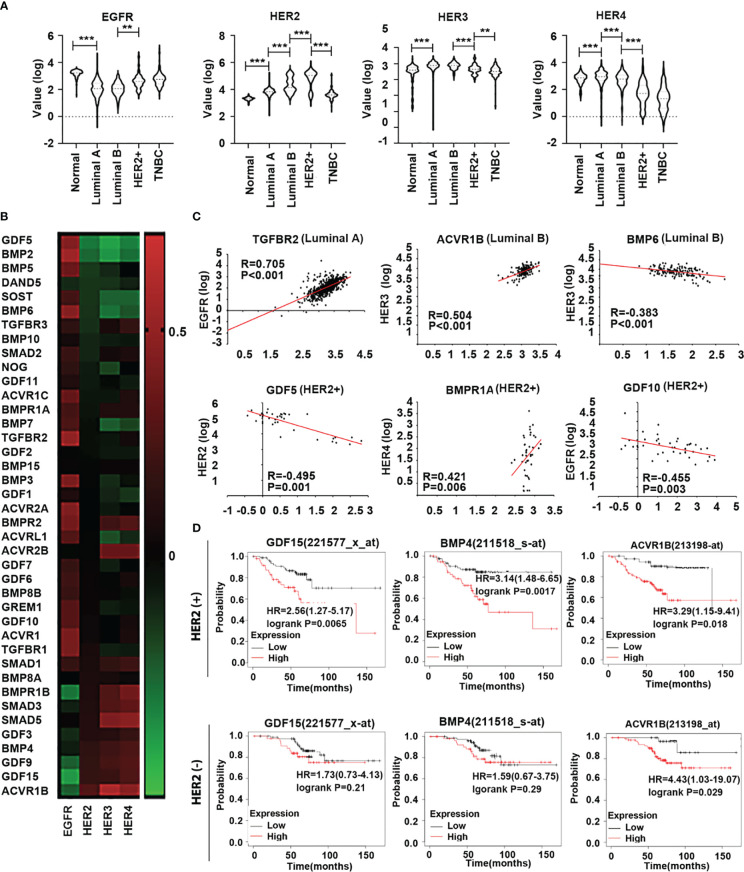
Expression of HER family in different subtypes of breast cancer. **(A)** Violin plots are shown in the TCGA dataset, the distribution of HER expression in normal tissue (n=112) and different subtypes of BRCA, including Luminal A (n=667), Luminal B (n=155), HER2+(n=41) and TNBC (n=179). **(B)** The heatmap shows the correlation between BMPs/BMPRs/BMP antagonists with HER family in breast cancer in the TCGA. **(C)** Scatterplots exhibit the gene expression level of significant BMPs/BMPRs/BMP antagonists in correlation with HER family in different BRCA subtypes. **(D)** Overall survival was analysed with online Kaplan-Meier plotter survival analysis (Http://kmplot/analysis) of specific BMPs which were highly associated with HER2 in HER2 positive tumours. **Represents P<0.01, ***represents P<0.001.

In addition to HER2, emerging evidence has shown that other members of the HER family are involved in breast cancer (16). Analyses of HERs in different subtypes of the disease in the TCGA cohort showed that HER2 was relatively higher in both Luminal B and HER2 positive subtypes. EGFR transcripts were higher in the tumours which lacked ER and PR, namely the HER2 positive and TNBC tumours. HER3 and HER4 are more frequently expressed in Luminal subtype tumours and appear to be reduced in both HER2 positive and TNBC tumours (**Figure 3A**). The results showed that all the correlations between these genes and HER family were significant throughout the whole breast cancer cohort (**Figure 3B**). In specific subtypes, there were different correlations among BMPs and HERs. TGFBR2 was positively associated with EGFR in Luminal A subtype. In Luminal B, BMP6 was negatively correlated with HER3 while ACVR1B was positively correlated with HER3. GDF5, together with GDF10 were negatively correlated with HER2/EGFR, whereas BMPR1A was positively correlated with HER4 in HER2 positive subtype (**Figure 3C**). Elevated expressions of GDF15 and BMP4 were associated with poorer prognosis comparing the low expression cohort (122.64 months) in HER2 negative patients with HER2 positive group (P<0.01), which indicates that GDF15 and BMP4 tend to promote the progression of breast cancer in HER2 (+) patients. Higher expression of ACVR1B was associated with shorter survival in both HER2(+) and HER2 (-) cohorts (**Figure 3D**).

### 3.4 BMPs were associated with proliferation markers in breast cancers

**Figure 4:**
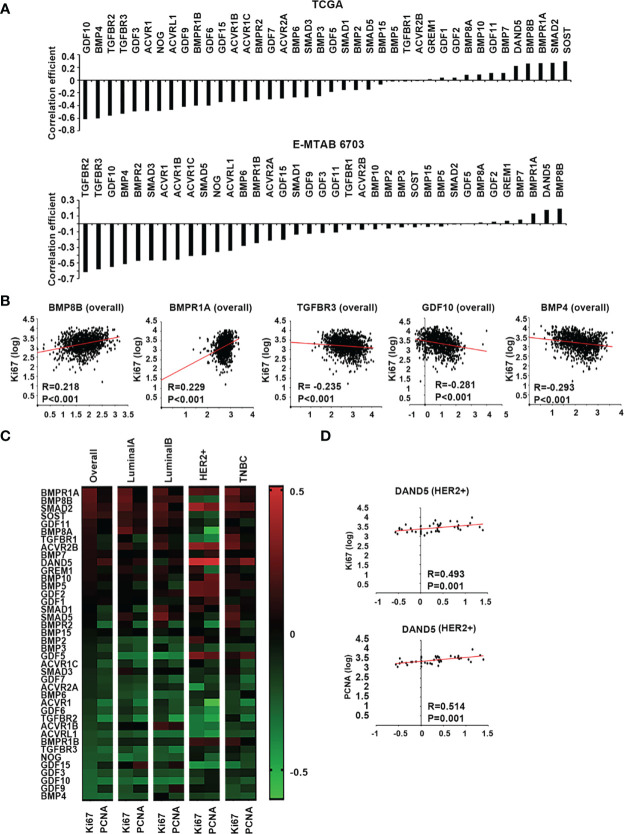
BMPs, BMP receptors and BMP antagonists in tumour growth of breast cancer. **(A)** Histograms of sum of correlation coefficient of BMPs, BMP receptors and BMP antagonists and cell proliferation markers in both TCGA (Cohort for discovery) and E-MTAB-6703 (Cohort of validation) datasets. **(B)** BMP8B, BMPR1A, TGFBR3, GDF10 and BMP4 expression were significantly correlated with Ki67 shown as scatter plots (based on the log value of gene expression). **(C)** Shown are heatmaps of correlation between BMPs, BMP receptors and BMP antagonists with cell proliferation markers in overall and different subtypes of BRCA. **(D)** The expression of DAND5 is shown in association with PCNA and Ki67 in HER2 positive subtype as scatter plots.

Two proliferation markers, Ki67 and PCNA, were employed in the Spearman correlation analyses to assess the relationship between BMPs and the proliferation status of tumours. Overall, the correlation coefficient, including both Ki67 and PCNA, showed that most BMPs, BMP receptors and antagonists were negatively correlated with the proliferation status of breast cancer with the leading genes: GDF10, BMP4, TGFBR2, TGFBR3 and GDF3, whilst DAND5, BMP8B, BMPR1A, Smad2 and SOST were positively correlated with proliferation markers in the TCGA breast cancer cohort. This was also evident in the E-MTAB-6703 cohort. The leading genes that were positively correlated with the proliferation markers, in both TCGA and E-MTAB-6703 cohorts, included BMP8B, DAND5 and BMPR1A, whilst TGFBR2, TGFBR3, GDF10, BMP4 and BMPR2 exhibited inverse correlation with proliferation (**Figure 4A**). Particularly, BMP8B and BMPR1A were highly positively correlated with Ki67, whilst BMP4, GDF10 and TGFBR3 exhibited inverse correlation with Ki67 (**Figure 4B**).

A further dissection of the correlation with proliferation markers, in different subtypes of the disease, revealed that certain BMPs may have a subtype specific role in tumour growth (**Figure 4C**). Compared to other subtypes, DAND5 had a significant positive correlation with both Ki67 and PCNA in HER2+ subtype (**Figure 4D**).

### 3.5 Correlation between BMPs and epithelial mesenchymal transition, MMPs in invasion of breast cancer

**Figure 5:**
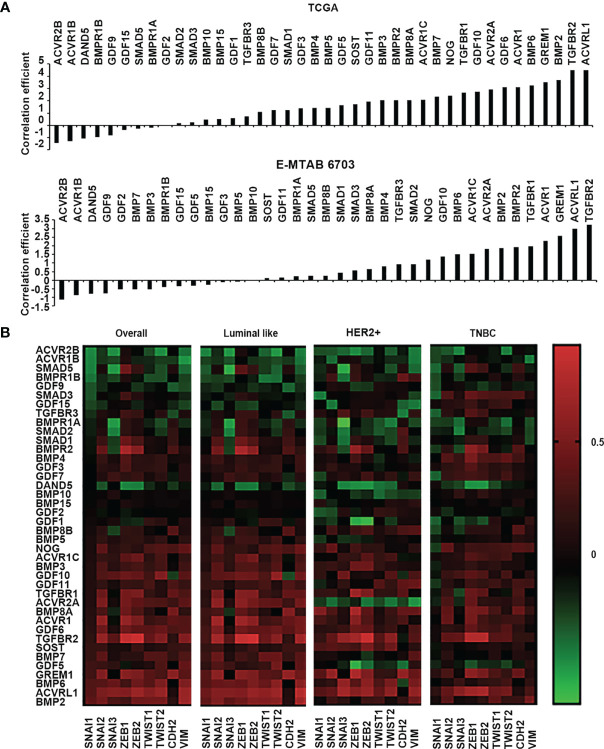
Correlation between BMPs, BMP receptors and BMP antagonists with EMT markers. **(A)** Histograms of sum of correlation coefficient of BMPs, BMP receptors and BMP antagonists and EMT markers (SNAI1, SNAI2, SNAI3, ZEB1, ZEB2, TWIST1, TWIST2, CDH2 and VIM) in both TCGA (Cohort for discovery) and E-MTAB-6703 (Cohort of validation) datasets. **(B)** Shown are heatmaps of correlation between BMPs, BMP receptors and BMP antagonists with EMT markers in overall and different subtypes of BRCA.

EMT is an essential cellular event for development, and also plays an important role in the tumourigenesis and disease progression of solid tumours (17). Invasion of cancerous cells can be regulated by BMPs in the tumour microenvironment through their coordination of EMT, MMPs, cytokines, and inflammatory cells etc. (18). Correlations with a panel of EMT markers, including Snail1, Snail2, Snail3, ZEB1, ZEB2, Twist1, Twist2, CDH2 (N-Cadherin) and VIM (vimentin) were analysed for BMPs in both TCGA and E-MTAB-6703 cohorts. Comparing the correlation with proliferation markers, most BMPs and BMP receptors were positively correlated with the EMT markers (**Figure 5A**), whilst only a few genes were inversely correlated with these markers. BMP6, GREM1, BMP2, TGFBR2 and ACVRL1 were the top five positively correlated genes with the EMT markers, whilst ACVR2B, ACVR1B, DAND5, BMPR1B and GDF9 were the top five negatively correlated genes observed in the TCGA cohort. In the E-MTAB-6703 cohort, TGFBR1, ACVR1, GREM1, ACVRL1 and TGFBR2 presented a remarkedly positive correlation with the EMT markers, whilst ACVR2B, ACVR1B, DAND5, GDF9 and GDF2 exhibited inverse correlation (**Figure 5A**). Correlation with the EMT markers was further analysed in different subtypes of the disease. BMP2 was associated with EMT markers in the Luminal subtype. BMP6 was positively correlated with EMTs in Luminal and HER2 positive breast cancer. BMP4 was positively correlated with EMT only in TNBC. On the other hand, both ACVR2B and DAND5 were negatively correlated with EMT in overall breast cancer. ACVR1B presented negative correlation in both Luminal and TNBC tumours but not in the HER2 positive tumours. BMPR1B was negatively associated with EMT markers only in Luminal subtype. Correlation between BMPR1A and EMT markers was evident in HER2 positive and TNBC breast cancers (**Figure 5B**).

MMPs (matrix metalloproteinases) influence tumour invasion and cancer cell migration which can be regulated by BMPs (4, 18). ACVRL1, GDF6, BMP8A, ACVR1 and GREM1 were seen as the top five genes which were positively correlated with the MMPs while ACVR1B, ACVR2B, GDF9, DAND5, BMPR1B were ranked as the top five being negatively correlated. ACVR1, ACVRL1, BMP8A, and GREM1 were also the leading genes positively correlated with MMPs in the E-MTAB-6703 cohort, while significantly inverse correlation was also evident for ACVR2B, ACVR1B and GDF9. In the TCGA dataset, GREM1 and ACVRL1 had significant positive correlations with most of the EMT markers and MMPs (**Supplementary Figure S2**).

### 3.6 BMP and angiogenesis in breast cancer

**Figure 6:**
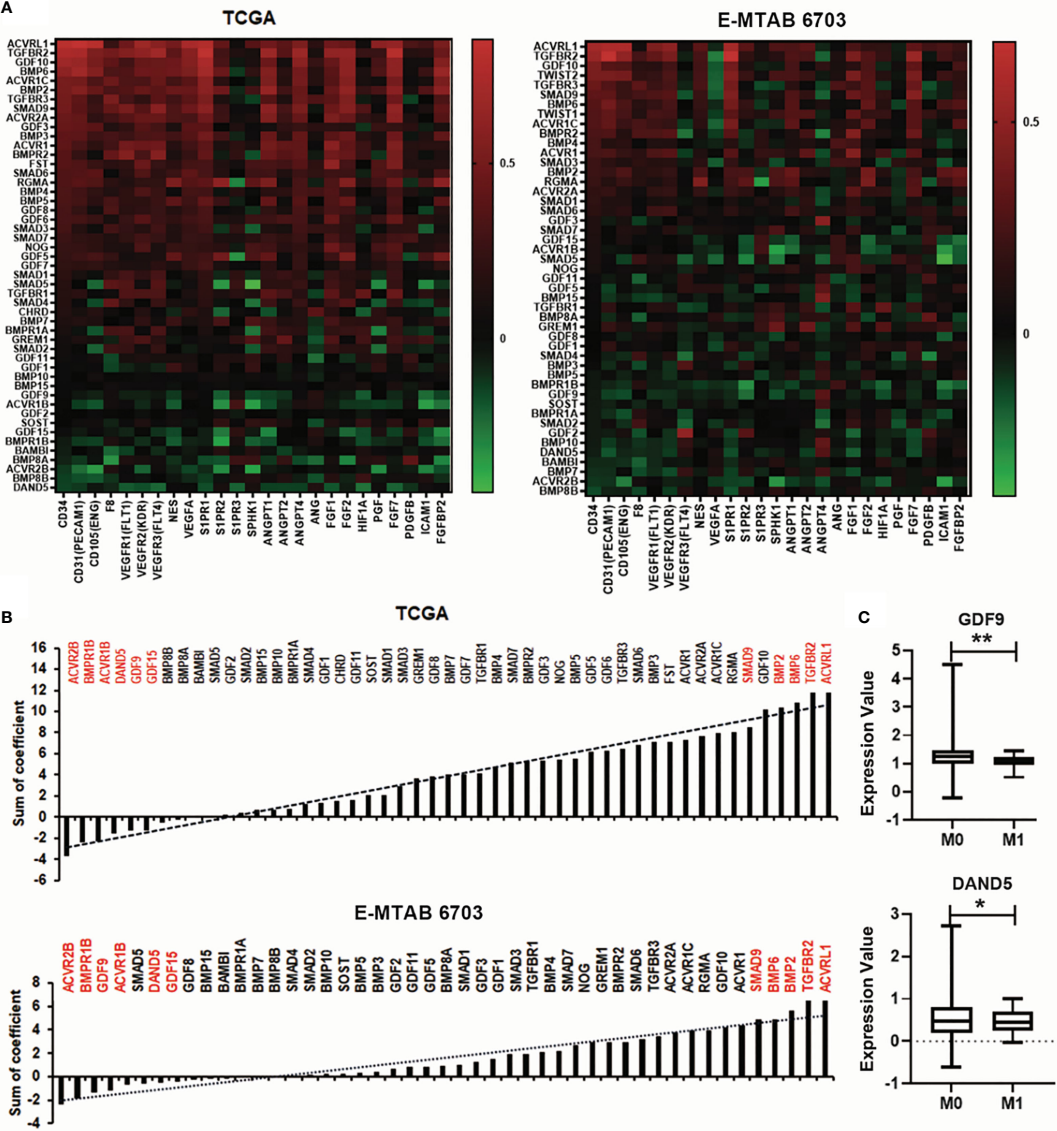
Association between BMPs and angiogenesis in BC. **(A)** Correlations between BMPs, BMP receptors and BMP antagonists with angiogenesis markers in both TCGA (cohort for discovery) and E-MTAB-6703 (cohort for validation), cohorts are shown as heatmaps. **(B)** Sum of correlation efficient of BMPs, BMP receptors and BMP antagonists and angiogenesis markers in both TCGA and E-MTAB-6703 cohorts are shown as histograms. Red labelling are the top genes positively/negatively correlated with angiogenic markers. **(C)** The expression of GDF9 and DAND5 in M staging in the TCGA cohort. *Represents P<0.05, **represents P<0.01.

New vasculatures formed in the tumours provide blood supply to bring in nutrients and also confer a route for cancer cells to spread. BMP can also regulate tumour associated angiogenesis (19). Correlation between BMPs and angiogenesis factors was analysed in both the TCGA and E-MTAB-6703 cohorts (**Figure 6A**). Regarding analysis of correlation coefficient values, the top five genes of ACVRL1, TGFBR2, BMP2, BMP6 and SMAD9 were positively correlated with angiogenesis markers. The expression of ACVR2B, BMPR1B, GDF9, ACVR1B, DAND5 and GDF15 were negatively associated with angiogenesis factors in both TCGA and E-MTAB-6703 cohorts (**Figure 6B**). The angiogenesis associated BMPs, BMP receptors and antagonists were further analysed for possible involvement in distant metastases. Reduced expression of GDF9 and DAND5 was observed in breast tumours which presented with distant metastasis (P<0.05) (**Figure 6C**).

### 3.7 Possible link between BMP and lymph angiogenesis in breast cancer

**Figure 7:**
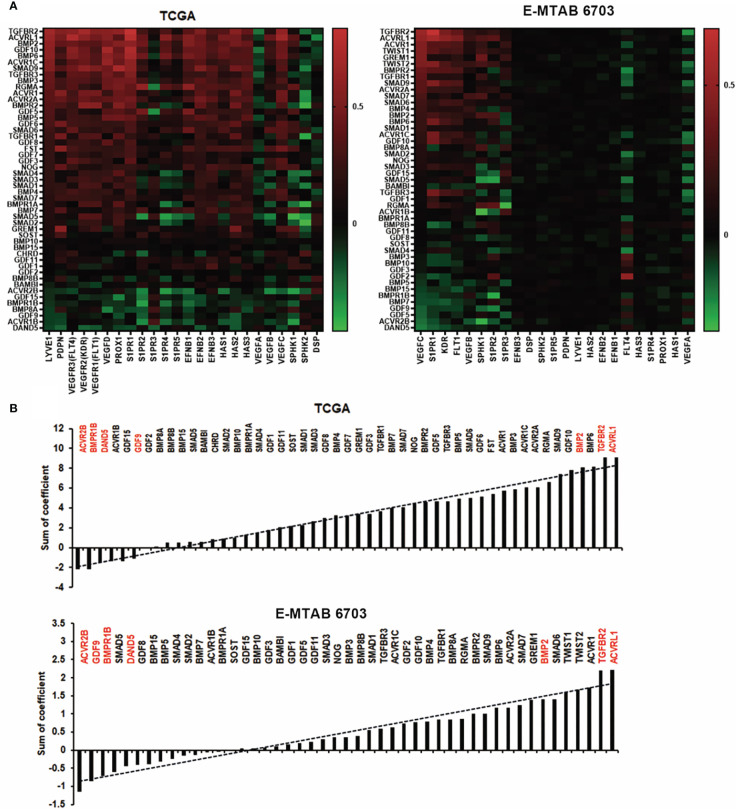
Association between BMPs and lymph angiogenesis in BC. **(A)** Shown are heatmaps of correlations between BMPs expression with lymph angiogenesis markers, in both TCGA and E-MTAB-6703 cohorts for discovery and validation. **(B)** Shown are histograms of sum of correlation efficient of BMPs, BMP receptors and BMP antagonists and lymph angiogenesis markers in both cohorts. Red labelling are the five leading genes positively/negatively correlated with lymph angiogenesis markers in both TCGA and E-MTAB-6703 cohorts.

Poor progression of cancer patients is mainly caused by metastasis. Tumour cell invasion and migration often happen in two ways, which comprise spreading through the blood vessels and lymphatic systems (20). We analysed the correlation among the BMPs with lymph angiogenesis factors such as LYVE1, VEGF, PDPN, PROX1, and S1PRs in both TCGA and E-MTAB-6703 cohorts (**Figure 7A**). It revealed that ACVRL1, TGFBR2 and BMP2 were positively correlated with lymph angiogenesis factors in discovery and validation cohorts (**Figure 7B**). One of the lymphatic-specific markers, LYVE1 (lymphatic vessel endothelial hyaluronan receptor 1) was first discovered in 1999 (21). It plays a critical role in identifying tumour-associated lymph angiogenesis (22). In the current study, we found that BMP2 was positively correlated with LYVE1 in the TCGA cohort. However, there was no obvious alteration of these BMP ligands and receptors in breast cancers that developed lymph node metastases (data not shown).

### 3.8 BMP and bone metastasis of breast cancer

**Figure 8:**
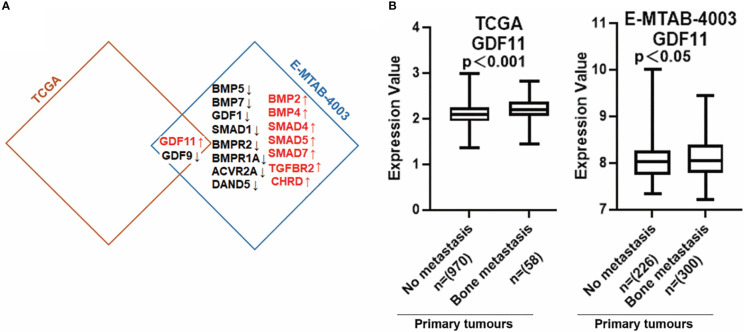
Aberrant expression of BMPs/BMPRs in bone metastasis of breast cancer. **(A)** Shown are the overlapping genes of GDF11 in both breast cancer with bone metastasis cohorts. **(B)** The expression of GDF11 in both BC cohorts with bone metastasis. Primary tumours tissue with no metastasis (TCGA n=970, E-MTAB-4003 n= 226) compared with (TCGA n=58, E-MTAB-4003 n= 300) primary tumours tissue with bone metastasis.

BMP ligands can activate osteolytic lesions which occur in breast cancer and BMP receptors which have been implicated in the progression of bone metastasis (8). Previous findings showed that Parathyroid hormone-related protein (PTHrP), receptor activator of nuclear factor-κB ligand (RANKL) and interleukin (IL)-11, IL-8, IL-6, etc, produced by metastatic cancer cells, are involved in osteolytic bone metastases (23). We found that BMPs were expressed differentially in primary tumours with bone metastasis of breast cancer. Low expression of GDF9 was observed in breast tumours which developed bone metastasis in both TCGA and E-MTAB-4003 cohorts, whilst increased GDF11 expression was seen in those primary tumours which developed bone metastasis. BMP2, BMP4, GDF11 and TGFBR2 had relatively higher levels in bone metastases of breast cancer while BMP5, BMP7, BMPR2, BMPR1A and ACVR2A presented lower expression in the bone metastases, in the E-MTAB-4003 dataset (**Figure 8A**). The increased expression of GDF11 in primary tumours with no metastasis compared with bone metastasis in BC has been clarified in both cohorts (**Figure 8B**).

### 3.9 Subtype specific expression profile of BMPs in breast cancer

**Figure 9:**
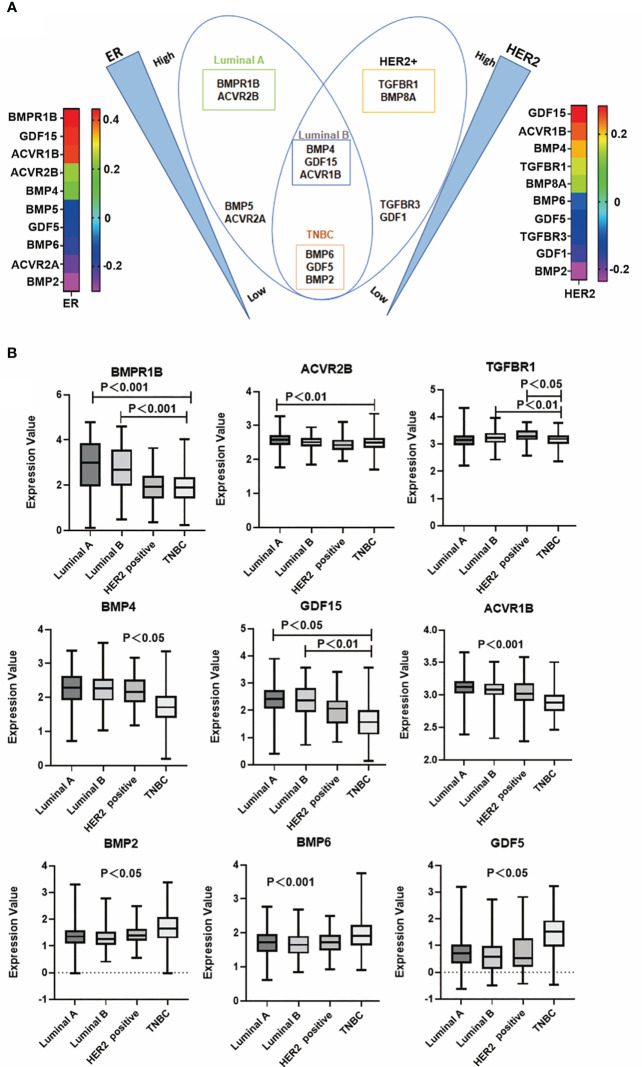
BMP and subtypes of breast cancer. **(A)** Butterfly plot shows that BMPs are most closely associated with ER and HER2 status in the E-MTAB-6703 cohort. **(B)** Aberrant expression of BMPR1B, ACVR2B, TGFBR1, BMP4, GDF15, ACVR1B, BMP2, BMP6 and GDF5 in Luminal A, Luminal B, HER2 positive and Triple negative breast cancer in the TCGA cohort, respectively.

To dissect the possible role of BMPs in different subtypes of breast cancer, correlation with markers for the subtyping was analysed. For example, BMPR1B, GDF15 and ACVR1B were positively correlated with ER. GDF15, ACVR1B and BMP4 were positively correlated with HER2. Butterfly-like plot shows subtype specific expression profile of the BMPs and BMP receptors according to their correlations with ER and HER2. BMPR1B and ACVR2B were positively correlated with ER in Luminal A subtype (ER+, HER2-) breast cancer. BMP4, GDF15 and ACVR1B were positively correlated with both ER and HER2 in Luminal B (ER+, HER2+) tumours. BMP8A and TGFBR1 were highly correlated with HER2 in the HER2 positive tumours. In addition to these, BMP2, BMP6 and GDF5 were highly expressed in the TNBC tumours compared with tumours of other subtypes (**Figure 9A**). Further analyses also showed that these specific genes expression in the TCGA cohort (**Figure 9B**).

### 3.10 Subtype specific association between BMPs and clinical outcomes in breast cancer

**Figure 10:**
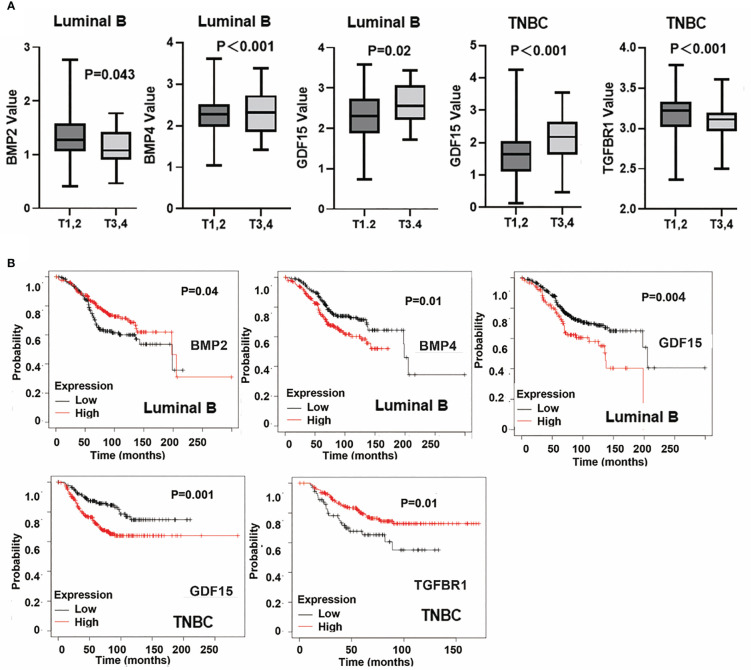
Specific BMPs in T staging and overall survival. **(A)** Expressions of BMP2, BMP4, GDF15 and TGFBR1 in T staging in Luminal A, Luminal B, HER2 positive and Triple negative breast cancer. **(B)** BMPs are significantly associated with patients’ overall survival in particular subtypes of breast cancer.

BMP2, BMP4, GDF15 and TGFBR1 were significantly correlated with T stage of breast cancer. Reduced BMP2 was evident in T3 and T4 tumours of the Luminal B subtype, in comparison with T1 and T2 tumours and the reduced expression was associated with poor overall survival in Luminal B subtype breast cancers (**Figures 10A, B**). Additionally, lower expression of TGFBR1 was observed in the locally advanced tumours (T3 and T4) in TNBC breast cancers, which was associated with poor OS in TNBC (**Figure 10B**). On the other hand, higher expression of BMP4 was observed in locally advanced Luminal B tumours and elevated GDF15 expression in those T3 and T4 tumours, of both Luminal B and TNBC subtypes, with poor OS (**Figures 10A, B**). No significant changes of these BMP ligands and receptors were evident in tumours which developed lymph node metastases and distant metastases (data not shown).

## 4 Discussion

In the current study, we analysed the expressions of BMPs and BMP receptors in breast cancer, using the TCGA RNA-sequencing dataset, and performed and validated in another cohort, which included paired breast tumours and adjacent normal tissues (GSE70951). It was shown that the expression of BMP8B was significantly increased in tumour compared with normal tissues, while BMP6 and ACVRL1 were expressed at lower levels. The findings are in line with previous studies (24, 25). In comparison with the expression profile of BMPs in normal breast tissues, shifts in the expression pattern were noticed for BMP2, BMP3, BMP5, BMP6, GDF5, GDF8, GDF10, ACVRL1, ACVR1, BMPR1A, TGFBR1, ACVR1C, ACVR2A, TGFBR2, TGFBR3 BMPR2 BMP8A, BMP8B, GDF9, GDF11, GDF15, ACVR1B, BMPR1B and GREM1.The role played by BMP8A is currently under investigation in a separate study at the host laboratory (unpublished data). The aberrant expression of BMP and their receptors are correlated to poor prognosis and progression in breast cancer patients. Lower expression levels of BMP2 and BMP6 were associated with poorer OS and RFS. On the contrary, high expression of TGFBR1 and GREM1 were significantly associated with poor OS, DMFS and RFS, except for the TGFBR1 which was not significantly associated with RFS. Decreased BMP6 enhances chemoresistance and promotes proliferation and invasion in MCF-7 cells (24, 26). Targeting the elevated expression of TGFBR1 with the inhibitor, EW-7195 can prevent metastasis of breast cancer cells to lungs by supressing Smad signalling (27). Elevated expression of GREM1 is correlated with poor survival in patients with ER negative breast cancer (28). However, high expression of BMP2 was found to be associated with microcalcification and poor prognosis (7). It suggests that different or contrasting roles may be played by BMPs in breast cancer which can be subtype specific, though certain BMP ligands, receptors and antagonists have been highlighted for their implication in the disease.

The involvement of oestrogen in the development and progression of breast cancer has been well established. ER positive tumours account for 70%-80% in breast cancer (29–31). The effect of oestrogen is largely mediated through two oestrogen receptors, ERα (ESR1) and ERβ (ESR2) (32). ER status is correlated with the prognosis of patients with breast cancer, being a key indicator for endocrine therapies. It has been shown that BMP6 and BMP7 can suppress oestrogen-promoted proliferation of the MCF-7 breast cancer cell line (33). Interestingly, oestrogen can compromise the BMP-induced inhibition by downregulating the expression of some BMP receptors, such as BMPR1A, BMPR1B, ACVR2A, and ACVR2B at transcription level (33). In the present study, ACVR1B and BMPR1B were found to be positively correlated with ESR1. Furthermore, we analysed the influence of ER on the expression of BMPs using a gene array data, in which ER was silenced in MCF-7 cells (GSE27473) (13). BMP2 was increased in the ER silenced MCF-7 cells. In addition to this direct impact on BMP2 by ER, oestrogen can also interfere with gene expression induced by BMP2 in MCF-7 cells (34). Reduced ACVR1B was observed in the ER silenced MCF-7 cells (GSE27473). However, increased expression of BMPR1B was exhibited in the ER silenced MCF-7 cells. It suggests that other regulatory mechanisms exist in the ER positive breast cancer cells to maintain the expression of BMPR1B. For instance, hepatocyte growth factor (HGF) is a cytokine being actively involved in the orchestrated crosstalk among different signalling pathways in cancer cells and exhibits a capability of regulating BMP and BMP receptors in cancer cells (35). BMP and its signalling can be regulated by oestrogen in breast cancer thus leading to diverse effects on cellular functions during disease progression, which helps to expand our understanding of the disease and tailor appropriate personalised treatment.

Amplified expression of HER2 occurs in 15% to 25% of breast cancer. HER2 targeted therapy involves HER2 receptor neutralising antibodies and inhibitors, including trastuzumab, pertuzumab, and lapatinib, which enhances the benefits from adjuvant therapies (36). The present study showed that EGFR was expressed relatively higher while HER4 was lower, in both HER2 positive and TNBC tumours. BMPR1A was positively correlated with HER2. BMP4, GDF15 and ACVR1B were expressed at higher levels in HER2 positive tumours which were correlated with poorer OS. To date, involvement of BMP signalling in HER2 positive breast cancer remains largely unknown. A recent study showed that GDF15 knockdown repressed invasion in HER2 positive breast cancer cells, indicating a therapeutic potential of targeting GDF15 (37). Taken together, these HER2 correlated BMPs and BMP receptors may be actively involved in the disease progression of HER2 positive tumours, which provokes further investigation to shed light on their subtype specific role.

BMPs can regulate proliferation of breast cancer cells, which can be ligand specific. For example, BMP2 and BMP6 inhibit cell proliferation whilst BMP7 promotes the proliferation of breast cancer cells (38–40). In the present study, an inverse correlation with the proliferation markers was evident for BMP ligands; GDF10, BMP4, GDF3 and GDF9, whilst BMP7 presented a weak but positive correlation with Ki67 only. Indeed, previous studies have shown that BMP4 can inhibit cell proliferation in breast cancer cell lines, such as MCF-7, SKBR3 (41) and MDA-MB-231 (42) and induces G1 cell cycle arrest in T-47D cells (43). This is in line with our current findings. In addition to the ligand specific regulation of proliferation, different receptors can also divert the impact on proliferation. A negative correlation with the proliferation markers was also seen for the receptors: TGFBR2, TGFBR3, ACVR1 and BMPR1B. A positive correlation was only seen in BMPR1A.

A recent study reported that O-GalNAcylation of up-regulated BMPR1A can promote cell growth in ER positive (MCF-7 and T-47D) cells, by inhibiting ERα expression (44). As a secreted BMP antagonist, DAND5 increased proliferation in MDA-MB-231 cells. Increased expression of DAND5 in breast cancer was associated with poor survival (45). The present study also revealed a positive correlation between DAND5 and the two proliferation markers (PCNA and Ki67) in HER2 positive tumours but not in other subtypes. It suggests that, in addition to the influence specified to certain BMP ligands and receptors, regulation of proliferation by BMPs in breast cancer can also be subtype specific. This adds another dimension for future exploration.

BMPs are well known as key molecules of involvement coordinating EMT during tumourigenesis and progression of malignancies. In the current study, a positive correlation with the EMT markers was evident for BMP2 and BMP6, while an adverse trend was evident for GDF9 and GDF15. In breast cancer, BMP2 can regulate EMT and stemness by upregulating CD44 and MMP11 *via* PI3K/AKT and Smad signalling pathways (46). High levels of BMP6 regulated E-cadherin by supressing deltaEF1 expression in MCF-7 cells. The expression of deltaEF1 is more likely associated with invasiveness in breast cancer cells (11). On the other hand, a high level of GDF9 was associated with good progression in breast tissues (47). Interestingly, a recent study showed that Diltiazem, a calcium channel blocker, was able to prevent EMT and reduce migration and invasion of TNBC cancer cells by up-regulating GDF15, with GDF15 itself having the similar inhibitory effect on invasiveness and EMT (48). This is in line with the inverse correlation, between GDF15 and EMT markers, observed in the present study. Therapeutic potential of utilising these BMPs is yet to be further investigated.

BMP receptors including ACVRL1, TGFBR2, ACVR1, ACVR2A and TGFBR1 were positively associated with invasiveness. A negative correlation was also seen for the receptors: ACVR2B, ACVR1B and BMPR1B. ACVRL1 induced metastasis and angiogenesis in mouse models and further demonstrated that ACVRL1 was involved in cell dissemination and tumour progression of breast cancer. Furthermore, overexpressed ACVRL1 is a factor of breast cancer patients with poor prognosis (49). TGFBR2 can be altered by USP11 (ubiquitin-specific peptidases 11) de-ubiquitination, which induced EMT in mammary epithelial cells *via* modulating TGF β signalling (50). TGFBR2, increased by histone acetylation, upregulated Snail and Slug expression, interfering with epithelial cell morphology (51). Dominant-negative mutant of ALK2 (DNALK2, ACVR1) reduces cell proliferation and metastasis *in vivo* and *in vitro* (52). An ALK5 inhibitor, vactosertib, can down-regulate the EMT markers (Vimentin, Snail, Slug, Twist) thus being considered as an option to prevent metastasis, when radiotherapy is applied to breast cancer patients (53).

BMP antagonists were also evaluated for their implication in EMT; NOG and GREM1 and presented a positive correlation with the EMT markers, whereas DAND5 showed an inverse correlation. High expression of Noggin has been implication in osteolytic bone metastases of breast cancer, reflecting its antagonism of BMP signalling (54, 55). Our recent study of Noggin in gastric cancer also revealed another dark side in cancers, as it facilitated proliferation of gastric cancer cells, by an upregulation of EGFR and its downstream signalling and also promoted EMT (56). However, the exact role of Noggin in breast cancer is yet to be fully evaluated. Similarly, GREM1 can also promote invasion of cancer cells by activating the EGFR pathway (57). Docosahexaenoic acid (DHA) can prevent GREM1-induced EMT in breast cancer cells by targeting ERK signalling (58). Taken together, these aforementioned BMP ligands, BMP receptors and antagonists present therapeutic potential for targeting the EMT events in breast cancer.

A recent literature review has highlighted that BMP2 enhanced angiogenesis through endothelial cells, whilst BMP9 and BMP10 inhibited cell growth and tumour angiogenesis in breast tumours (59). The present study showed that BMP ligands such as BMP2 and BMP6 are more likely to enhance angiogenetic metastasis but GDF9 and GDF15 are contradictory. Raida, Clement et al. noticed that BMP2 enhanced cell vasculature formation, by activating ID1 and VEGF through P38 signalling (60). BMP6 enhanced angiogenic responses with the regulation of Cox2 (cyclppxygenase2) in disease (61). BMP6 causes tumour angiogenesis and proliferation *via* stimulating IL-Iα in prostate cancer (62). BMP2/4/6 are the key molecules targeting VEGF on endothelial cells. BMP6 was found to mediate new vessel formation directly. Thus, BMP2/BMP6 may act as specific markers for anti-angiogenic targeted therapy (63). For certain BMP receptors, ACVRL1 and TGFBR2 are positively associated with angiogenic markers. Knockdown of TGFBR2 inhibits tumour angiogenesis by miR-204 modulation (64). On the contrary, ACVR2B, BMPR1B, ACVR1B and DAND5 are negatively correlated with angiogenetic factors. However, it has been reported that DAND5 can promote growth of vascular endothelial cells and angiogenesis *in vitro* and *in vivo* (45). A disease specific role may be played by DAND5 in the tumour associated angiogenesis of breast cancer, which is yet to be investigated. In addition to angiogenesis, we also evaluated the impact of BMPs on the lymphangiogenesis, which is also importance for dissemination of breast cancer cells. The present study showed that BMP2, BMP6, GDF10 and BMP3 are positively correlated with lymphangiogenetic markers, whilst GDF9 is negatively correlated with them. One review mentioned that BMP-SMAD signalling is highly connected in the vascular and lymphatic endothelial systems of cardiac diseases (65). Until now, BMP in lymphangiogenesis is still largely a mystery in breast cancer. Furthermore, BMP receptors which are positively correlated with lymphangiogenetic markers are as follows: ACVRL1, TGFBR2, ACVR2A and ACVR1. A negative correlation was seen in ACVR2B, BMPR1B and ACVR1B. ACVRL1 would be a key BMP receptor with breast cancer angiogenesis and lymphangiogenesis. One study exhibited that ALK1 targeted therapy can inhibit angiogenesis and clinical trials showed potential ways to improve end stage cancer patients’ survival with ALK1 inhibitors (66, 67). However, a previous paper reported ALK-1 (ACVRL1) signals inhibit LV (Lymphatic vessels) formation (68). The potential of targeting these BMPs, receptors and antagonists for currently incurable breast cancer warrants further exploration.

Bone is the most common site of metastasis in BC patients, with up to 70% of advanced BC patients developing skeletal metastases (69). BMP2 can regulate maturation and differentiation of osteoclastic cells through the PI3K/AKT pathway (7). Reduced expression of BMP4 supressed osteoclastic cell differentiation with regulation of Fam20C in breast cancer. Fam is a phosphoprotein which may have an impact on bone resorption (70). Depletion of TGFBR2 is associated with metastasis to lungs and bone (71). Soluble TGFBR2 with human immunoglobulin Fc (Stgfbr2Fc) can prevent bone osteolysis (72). Our recent study has shown that GDF11 is a promising molecule involved in the predisposition of osteolytic bone metastasis in breast cancer (73). Knockdown of BMPR1A evades osteolytic bone metastasis by repressing RANKL expression by way of P38 pathway in MDA-MB-231 cells and in mice (74). However, this is not truly consistent with our finding that lower levels of BMPR1A occur in primary tumour with bone metastasis group compared to primary tumour without distant metastasis in the E-MTAB-4003 cohort.

In the present study, deregulated BMP and BMP receptors were revealed in certain subtypes of breast cancer. Higher expression of BMPR1B and ACVR1B were shown in Luminal A and Lumina B subtypes compared to in TNBC. The experimental evidence revealed that BMPR1B expression affected tumour growth in ER positive carcinomas (75). Decreased expression of BMPR1B promotes proliferation in MDA-MB-231 cells (76). Overexpressed CYP (Cytochrome P450) 2J2 can activate BMPR1B expression to increase migration in MDA-MB-468 cells (77). Implication of ACVR2B in breast cancer remains unknown. One study in colorectal cancer reported that ACVR2B once bound to BMP3 thereafter resistant cell apoptosis *via* Smad-dependent signalling (78). Overexpression of GDF15 facilities cell cycle in S phase in BT474 cells derived from Luminal B subtype (37). This is consistent with our finding that GDF15 is expressed higher in T3,4 staging compared to T1,2 with poorer OS. Data analysis clearly suggested lower expression of TGFBR1 in later staging of breast cancer with poor progression in TNBC. Safina et al. reported TGFBR1 enhanced tumour invasion and new blood vessel formation by stimulating MMP9 in MDA-MB-231 cells (79). However, the subtype specific involvement and functional impact of these BMPs and BMP receptors, in breast cancer, are still largely unknown.

## 5 Conclusion

In summary, a shift in the expression pattern of BMPs in breast cancer, including increased BMP8A, BMPR1B and decreased BMP6, BMP7, ACVR1C, TGFBR2, TGFBR3 and BMPR2 presented a subtype specific role. In four main subtypes of breast cancer, elevated expression of BMPR1B and ACVR2B were seen in Luminal A subtype, while BMP4, GDF15 and ACVR1B were higher in Luminal B, TGFBR1 and BMP8A were higher in HER2 positive, BMP2, BMP6 and GDF5 were higher in TNBC. It suggests that these BMPs and receptors may be actively engaged in the disease progression and distant metastasis through a regulation of proliferation, invasion and EMT, which warrants further investigation to dissect their subtype specific role, to present a proof of concept for personalised target therapy.

## Data availability statement

The original contributions presented in the study are included in the article/**Supplementary Material**. Further inquiries can be directed to the corresponding author.

## Author contributions

LY and WJ conceived the study. ML, ZF, and LS sourced the dataset and statistical analysis, all participated in manuscript preparation. All authors have read and agreed to the published version of the manuscript. All authors contributed to the article.
