# Supplementary material for: Aberrant expression of bone morphogenetic proteins in the disease progression and metastasis of breast cancer
Source: Front Oncol. 2023 Jun 2;13:1166955. doi: 10.3389/fonc.2023.1166955 (PMC10272747; doi:10.3389/fonc.2023.1166955)
Supplement: Supplementary file 1 [file Table_1.docx]

**Supplementary Table 1. Different expressions of BMP and BMP receptors in the TCGA_BRCA cohort.**

| **Gene name** | **Normal** | **Tumour** | **Absolute change** | **P value** |
| --- | --- | --- | --- | --- |
| BMP2 | 173.044 | 22.24595 | -150.798 | 8.69E-17 |
| BMP3 | 20.872 | 1.1569 | -19.7151 | 0.031104 |
| BMP5 | 42.0893 | 1.4084 | -40.6809 | 2.5E-16 |
| BMP6 | 283.3348 | 61.15305 | -222.182 | 2.47E-19 |
| BMP8A | 39.2088 | 229.2546 | 190.0458 | 2.43E-20 |
| BMP8B | 50.12855 | 81.2136 | 31.08505 | 2.81E-07 |
| GDF5 | 32.53205 | 4.8789 | -27.6532 | 6.01E-06 |
| GDF8 | 11.4777 | 2.6067 | -8.871 | 0.005866 |
| GDF9 | 15.1372 | 15.75025 | 0.61305 | 0.015567 |
| GDF10 | 245.3799 | 10.0427 | -235.337 | 1.86E-19 |
| GDF11 | 96.457 | 124.34 | 27.88295 | 4.82E-05 |
| GDF15 | 55.85495 | 134.3586 | 78.5036 | 0.000102 |
| ACVRL1 | 955.7668 | 417.6519 | -538.115 | 8.61E-17 |
| ACVR1 | 1195.476 | 1012.663 | -182.813 | 0.000172 |
| BMPR1A | 903.8981 | 626.515 | -277.383 | 5.96E-14 |
| ACVR1B | 1085.501 | 1219.895 | 134.3942 | 0.000112 |
| TGFBR1 | 1783.831 | 1634.561 | -149.27 | 0.584262 |
| BMPR1B | 90.4196 | 509.1311 | 418.7115 | 1.5E-06 |
| ACVR1C | 753.9357 | 26.69535 | -727.24 | 1.56E-10 |
| TGFBR2 | 9412.066 | 2006.362 | -7405.7 | 2E-32 |
| TGFBR3 | 5696.476 | 790.1188 | -4906.36 | 9.62E-44 |
| BMPR2 | 3873.094 | 3265.842 | -607.252 | 0.0003 |
| ACVR2A | 578.8822 | 319.1904 | -259.692 | 9.64E-27 |
| GREM1 | 301.7022 | 1011.123 | 709.421 | 5.23E-08 |
