# Supplementary material for: Aberrant expression of bone morphogenetic proteins in the disease progression and metastasis of breast cancer
Source: Front Oncol. 2023 Jun 2;13:1166955. doi: 10.3389/fonc.2023.1166955 (PMC10272747; doi:10.3389/fonc.2023.1166955)
Supplement: Supplementary file 2 [file Table_2.docx]

**Supplementary Table 2.1 Expression of BMP in breast cancer.**

|  | **Normal**  **(n=43)** | **Adjacent**  **(n=47)** | **Tumour**  **(n=47)** |  |
| --- | --- | --- | --- | --- |
| **BMP3** | 16.33(16.06-16.56)^*^ | 16.79(16.55-16.98) | 16.64(16.35-16.93) |  |
| **BMP4** | 6.80(6.74-6.89)^*^ | 6.84(6.77-6.91)^∆^ | 6.93(6.84-7.07) |  |
| **BMP5** | 6.70(6.62-6.76) | 6.72(6.66-6.80) | 6.73(6.66-6.84) |  |
| **BMP6** | 13.80(13.22-14.35)^*^ | 14.60(14.07-14.94)^∆^ | 13.29(11.94-13.87) |  |
| **BMP8B** | 7.35(6.98-7.89)^*^ | 7.45(7.14-8.07) | 7.79(7.25-8.60) |  |
| **BMP10** | 13.35(13.11-13.51)^*^ | 13.06(12.86-13.28) | 12.82(12.43-13.13) |  |
| **BMP15** | 8.98(7.38-9.95)^*^ | 8.70(7.70-9.49)^∆^ | 11.79(10.26-12.48) |  |
| **GDF2** | 7.79(7.08-8.81)^*^ | 8.42(7.42-9.25) | 8.70(7.64-9.61) |  |
| **GDF3** | 13.63(13.24-13.93)^*^ | 13.13(12.86-13.61) | 13.07(12.52-13.47) |  |
| **GDF5** | 6.84(6.65-7.02)^*^ | 6.66(6.59-6.74)^∆^ | 7.09(6.86-7.58) |  |
| **GDF6** | 16.52(15.97-17.01)^*^ | 17.08(16.73-17.33) | 16.92(16.57-17.24) |  |

Note: Shown are median of each gene expressed in a cohort of breast cancer in comparison with normal and adjacent breast tissues derived from a dataset (GSE70951). * Presents P<0.05 vs Normal, ∆ represents p<0.05 vs Adjacent.

**Supplementary Table 2.2 Expression of BMP Receptor in breast cancer.**

|  | **Normal**  **(n=43)** | **Adjacent**  **(n=47)** | **Tumour**  **(n=47)** |
| --- | --- | --- | --- |
| **ACVRL1** | 12.03(11.56-12.47)^*^ | 11.95(11.29-12.21)^∆^ | 10.32(9.95-11.16) |
| **ACVR1B** | 6.58(6.52-6.66) | 6.61(6.53-6.65) | 6.62(6.57-6.70) |
| **ACVR1C** | 6.66(6.61-6.79)^*^ | 6.74(6.70-6.80) | 6.75(6.69-6.81) |
| **BMPR1A** | 6.92(6.78-7.02) | 7.19(7.01-7.37)^∆^ | 6.90(6.77-7.02) |
| **BMPR1B** | 7.03(6.93-7.23) | 7.08(6.92-7.16) | 6.99(6.93-7.10) |
| **TGFBR2** | 6.78(6.70-6.96) | 6.84(6.72-6.94) | 6.74(6.70-6.83) |

Note: Shown are median of each gene expressed in a cohort of breast cancer in comparison with normal and adjacent breast tissues derived from a dataset (GSE70951). * Presents p<0.05 vs Normal, ∆ represents p<0.05 vs Adjacent.

**Supplementary Table 2.3 Expression of BMP Antagonists in breast cancer.**

|  | **Normal**  **(n=43)** | **Adjacent**  **(n=47)** | **Tumour**  **(n=47)** |
| --- | --- | --- | --- |
| **TWIST1** | 7.04(6.78-7.41)* | 7.97(7.64-8.34) | 8.16(7.76-9.19) |
| **GREM1** | 9.21(9.00-9.36)^*^ | 8.97(8.76-9.20) | 9.02(8.83-9.17) |
| **NOG** | 7.13(6.97-7.39) | 7.26(7.15-7.50) | 7.30(7.02-7.65) |

Note: Shown are median of each gene expressed in a cohort of breast cancer in comparison with normal and adjacent breast tissues derived from a dataset (GSE70951). * represents p<0.05 vs Normal, ∆ represents p<0.05 vs Adjacent.
