# Supplementary material for: Aberrant expression of bone morphogenetic proteins in the disease progression and metastasis of breast cancer
Source: Front Oncol. 2023 Jun 2;13:1166955. doi: 10.3389/fonc.2023.1166955 (PMC10272747; doi:10.3389/fonc.2023.1166955)
Supplement: Supplementary file 3 [file Table_3.docx]

**Supplementary Table 3.1 BMP expression and overall survival of BC patients (the KMplot cohort).**

| **Gene Median cutoff** | | | | | | | | **Median OS (months)** | | | **P** |
| --- | --- | --- | --- | --- | --- | --- | --- | --- | --- | --- | --- |
|  |  |  |  |  |  |  |  | **Low expression (months)** | **High expression (months)** | |  |
| **BMP2** |  | 30 43 | | | | | | 129.1 | 219.77 | | 0.02 |
| **BMP3** |  | | | | 1 3 | | | 124.53 | 142.23 | | 0.02 |
| **BMP4** |  | 199 330 | | | | | | 124.53 | 148.53 | | 0.13 |
| **BMP5** | | | | | |  | 6 2 | 108.73 | 131.5 | ＜0.001 | |
| **BMP6** | |  | | | 58 32 | | | 108.73 | 142.23 | ＜0.001 | |
| **BMP7** | | |  | | 104 223 | | | 129.1 | 131.97 | | 0.22 |
| **BMP8A** |  | | | 26 15 | | | | 215.2 | 122.3 | | 0.01 |
| **BMP8B** |  | 142 168 | | | | | | 87.87 | 64.23 | | 0.001 |
| **BMP10** |  | 0 0 | | | | | | 215.2 | 93.27 | ＜0.001 | |
| **BMP15** |  | 0 0 | | | | | | 148.53 | 98.83 | ＜0.001 | |
| **GDF1** |  | 7 10 | | | | | | 129.1 | 142.23 | | 0.049 |
| **GDF2** |  | 0 0 | | | | | | 215.2 | 93.27 | ＜0.001 | |
| **GDF3** |  | 3 4 | | | | | | 148.53 | 113.93 | | 0.01 |
| **GDF5** |  | 5 4 | | | | | | 113.63 | 142.23 | | 0.004 |
| **GDF6** |  | 12 22 | | | | | | 131.97 | 122.3 | | 0.12 |
| **GDF7** |  | 6 4 | | | | | | 124.53 | 131.37 | | 0.21 |
| **GDF9** |  | 27 31 | | | | | | 148.53 | 116.4 | | 0.41 |
| **GDF10** |  | 11 10 | | | | | | 129.1 | 131.97 | | 0.06 |
| **GDF11** |  | 220 304 | | | | | | 131.97 | 115.37 | | 0.11 |
| **GDF15** |  | 236 440 | | | | | | 142.23 | 116.4 | | 0.29 |

Note: Shown is overall survival of each gene expressed in breast cancer patients in RNA sequences (n=1090) derived from Kaplan-Meier Plot survival analysis (http://kmplot.com).

**Supplementary Table 3.2 BMP receptor and overall survival of BC patients (the KMplot cohort).**

| **Gene Median cutoff** | | | **Median OS (months)** | | **P** |
| --- | --- | --- | --- | --- | --- |
|  |  |  | **Low expression (months)** | **High expression (months)** |  |
| **ACVRL1** |  | 523 408 | 148.53 | 122.3 | 0.23 |
| **ACVR1** |  | 1107 1369 | 148.53 | 113.93 | 0.08 |
| **BMPR1A** |  | 848 865 | 148.53 | 115.4 | 0.10 |
| **ACVR1B** |  | 1525 1881 | 131.97 | 129.1 | 0.15 |
| **TGFBR1** |  | 1774 1727 | 215.2 | 115.37 | 0.003 |
| **BMPR1B** |  | 383 301 | 130.87 | 131.37 | 0.24 |
| **ACVR1C** |  | 24 31 | 130.87 | 131.37 | 0.15 |
| **TGFBR2** |  | 2213 2310 | 131.37 | 122.3 | 0.23 |
| **TGFBR3** |  | 687 797 | 129.1 | 131.97 | 0.1 |
| **BMPR2** |  | 3211 3420 | 215.2 | 115.37 | 0.15 |
| **ACVR2B** |  | 447 455 | 148.53 | 131.37 | 0.05 |
| **ACVR2A** |  | 360 320 | 130.87 | 142.23 | 0.06 |

Note: Shown is overall survival of each gene expressed in breast cancer patients in RNA sequences (n=1090) derived from Kaplan-Meier Plot survival analysis (http://kmplot.com).

**Supplementary Table 3.3 Smad expression and overall survival of BC patients (the KMplot cohort).**

|  |  | | |  | **Median OS (months)** | | **P** |
| --- | --- | --- | --- | --- | --- | --- | --- |
| **Gene** |  | | **Median cutoff** | | **Low expression (months)** | **High expression (months)** |  |
| **SMAD1** |  | 644 482 | | | 219.77 | 122.3 | 0.22 |
| **SMAD2** |  | 2488 2403 | | | 215.2 | 122.3 | 0.15 |
| **SMAD3** |  | 1449 1178 | | | 131.5 | 130.87 | 0.11 |
| **SMAD4** |  | 1657 1628 | | | 215 .2 | 116.4 | 0.16 |
| **SMAD5** |  | 1839 1972 | | | 215.2 | 115.37 | 0.04 |
| **SMAD6** |  | 166 124 | | | 248.5 | 129.1 | 0.08 |
| **SMAD7** |  | 843 855 | | | 148.53 | 122.3 | 0.23 |
| **SMAD9** |  | 94 102 | | | 131.37 | 142.23 | 0.28 |

Note: Shown is overall survival of each gene expressed in breast cancer patients in RNA sequences (n=1090) derived from Kaplan-Meier Plot survival analysis (http://kmplot.com).

**Supplementary Table 3.4 BMP antagonists and overall survival of BC patients (the KMplot cohort).**

|  |  | | |  | **Median OS (months)** | | | **P** |
| --- | --- | --- | --- | --- | --- | --- | --- | --- |
| **Gene** |  | | **Median cutoff** | | **Low expression (months)** | **High expression (months)** | |  |
| **NOG** |  | 4 4 | | | 148.53 | 115.4 | 0.052 | |
| **GREM1** |  | 946 704 | | | 248.5 | 115.4 | 0.046 | |
| **DAND5** |  | 5 4 | | | 113.93 | 219.77 | 0.007 | |
| **SOST** |  | 0 0 | | | 148.53 | 124.53 | 0.02 | |

Note: Shown is overall survival of each gene expressed in breast cancer patients in RNA sequences (n=1090) derived from Kaplan-Meier Plot survival analysis (http://kmplot.com).
