# Supplementary material for: Aberrant expression of bone morphogenetic proteins in the disease progression and metastasis of breast cancer
Source: Front Oncol. 2023 Jun 2;13:1166955. doi: 10.3389/fonc.2023.1166955 (PMC10272747; doi:10.3389/fonc.2023.1166955)
Supplement: Supplementary file 4 [file Table_4.docx]

**Supplementary Table 4.1 BMP expression and overall survival of BC patients (the KMplot cohort).**

| **Gene Affy ID Median cutoff** | | | **Median OS (months)** | | **p** |  |
| --- | --- | --- | --- | --- | --- | --- |
|  |  |  | **Low expression (months)** | **High expression (months)** |  |  |
| **BMP2** | 205289_at | 52 36 | 68.4 | 121.2 | 0.002 |  |
| **BMP3** | 208244_at | 40 80 | 90 | 118.62 | 0.12 |  |
| **BMP4** | 211518_s_at | 22 49 | 115 | 80 | 0.04 |  |
| **BMP5** | 205431_s_at | 111 79 | 118.82 | 90 | 0.2 |  |
| **BMP6** | 206176_at | 157 227 | 84 | 143 | 0.009 |  |
| **BMP7** | 209590_at | 144 81 | 125.92 | 89.04 | 0.11 |  |
| **BMP8A** | 220203_at | 78 132 | 99.6 | 84 | 0.17 |  |
| **BMP8B** | 207865_s_at | 48 29 | 81 | 108 | 0.37 |  |
| **BMP10** | 208292_at | 58 45 | 89.04 | 108 | 0.25 |  |
| **BMP15** | 221332_at | 113 189 | 90 | 120 | 0.09 |  |
| **GDF1** | 206397_x_at | 19 30 | 121.2 | 69.4 | ＜0.001 | |
| **GDF2** | 221136_at | 68 71 | 106.8 | 86 | 0.13 |  |
| **GDF3** | 220053_at | 91 60 | 77.4 | 108 | 0.17 |  |
| **GDF5** | 206614_at | 63 54 | 130 | 82 | 0.04 |  |
| **GDF8** | 207145_at | 15 20 | 85.2 | 110.04 | 0.08 |  |
| **GDF9** | 221314_at | 152 90 | 70.68 | 118.03 | 0.009 |  |
| **GDF10** | 206159_at | 82 52 | 81.6 | 118.82 | 0.02 |  |
| **GDF11** | 216854_at | 12 11 | 115 | 84 | 0.22 |  |
| **GDF15** | 221577_x_at | 254 359 | 118.03 | 69.73 | ＜0.001 | |

Note: Shown is overall survival of each gene expressed in breast cancer patients in RNA chips (n=1042) derived from Kaplan-Meier Plot survival analysis (http://kmplot.com).

**Supplementary Table 4.2 BMP receptor and overall survival of BC patients (the KMplot cohort).**

| **Gene Affy ID Median cutoff** | | | **Median OS (months)** | | **p** |
| --- | --- | --- | --- | --- | --- |
|  |  |  | **Low expression (months)** | **High expression (months)** |  |
| **ACVRL1** | 226950_at | 196 252 | 74.4 | 121.2 | 0.22 |
| **ACVR1** | 203935_at | 1043 826 | 76.8 | 115 | 0.05 |
| **BMPR1A** | 213578_at | 660 850 | 115 | 69.6 | 0.02 |
| **ACVR1B** | 213198_at | 1168 1251 | 81.87 | 120 | 0.05 |
| **TGFBR1** | 224793_s_at | 1913 2492 | 95.04 | 63.52 | 0.04 |
| **BMPR1B** | 229975_at | 260 1971 | 68.4 | 135.84 | 0.004 |
| **ACVR1C** | 1563182_at | 55 39 | 138 | 169.2 | 0.06 |
| **TGFBR2** | 208944_at | 1642 1494 | 67.56 | 136.8 | <0.001 |
| **TGFBR3** | 226625_at | 802 635 | 56.28 | 135.84 | <0.001 |
| **BMPR2** | 225144_at | 1753 1589 | 138 | 169.2 | <0.001 |
| **ACVR2B** | 236126_at | 308 274 | 68.4 | 90 | 0.09 |
| **ACVR2A** | 228416_at | 331 253 | 69.73 | 89.04 | 0.20 |

Note: Shown is overall survival of each gene expressed in breast cancer patients in RNA chips (n=1042) derived from Kaplan-Meier Plot survival analysis (http://kmplot.com).

**Supplementary Table 4.3 Smad expression and overall survival of BC patients (the KMplot cohort).**

|  |  |  | **Median OS (months)** | | **P** |
| --- | --- | --- | --- | --- | --- |
| **Gene** | **Affy ID** | **Median cutoff** | **Low expression (months)** | **High expression (months)** |  |
| **SMAD1** | 208693_s_at | 2623 3266 | 136.8 | 53.04 | <0.001 |
| **SMAD2** | 226563_at | 599 648 | 85.2 | 78 | 0.27 |
| **SMAD3** | 218284_at | 533 446 | 70.68 | 121.2 | 0.01 |
| **SMAD4** | 235725_at | 447 432 | 68.4 | 90 | 0.27 |
| **SMAD5** | 225223_at | 1120 1170 | 66.72 | 106.8 | 0.04 |
| **SMAD6** | 207069_s _at | 221 282 | 123.6 | 56.93 | <0.001 |
| **SMAD7** | 204790_at | 650 838 | 90 | 130 | 0.11 |
| **SMAD9** | 227719_at | 273 293 | 63.96 | 108 | 0.02 |

Note: Shown is overall survival of each gene expressed in breast cancer patients in RNA chips (n=1042) derived from Kaplan-Meier Plot survival analysis (http://kmplot.com) .

**Supplementary Table 4.4 BMP antagonists and overall survival of BC patients (the KMplot cohort).**

|  |  |  | **Median OS (months)** | | **p** |
| --- | --- | --- | --- | --- | --- |
| **Gene** | **Affy ID** | **Median cutoff** | **Low expression (months)** | **High expression (months)** |  |
| **NOG** | 231798_at | 24 36 | 108 | 63.83 | 0.02 |
| **FLRG** | 203592_at | 344 397 | 86 | 143 | 0.03 |
| **TSG** | 219201_s_at | 153 183 | 108 | 90 | 0.24 |
| **GREM1** | 218469_at | 705 1013 | 115 | 74.4 | 0.02 |
| **DAN** | 203905_at | 1310 1681 | 90 | 143 | 0.03 |
| **PRDC** | 220794_at | 34 72 | 90 | 135.88 | 0.09 |
| **SOST** | 223869_at | 12 9 | 63.6 | 97.2 | 0.09 |
| **DAND5** | 1562772_a_at | 46 30 | 66 | 90 | 0.16 |

Note: Shown is overall survival of each gene expressed in breast cancer patients in RNA chips (n=1042) derived from Kaplan-Meier Plot survival analysis (http://kmplot.com).
