# Supplementary figures and images for: Aberrant expression of bone morphogenetic proteins in the disease progression and metastasis of breast cancer
Source: Front Oncol. 2023 Jun 2;13:1166955. doi: 10.3389/fonc.2023.1166955 (PMC10272747; doi:10.3389/fonc.2023.1166955)

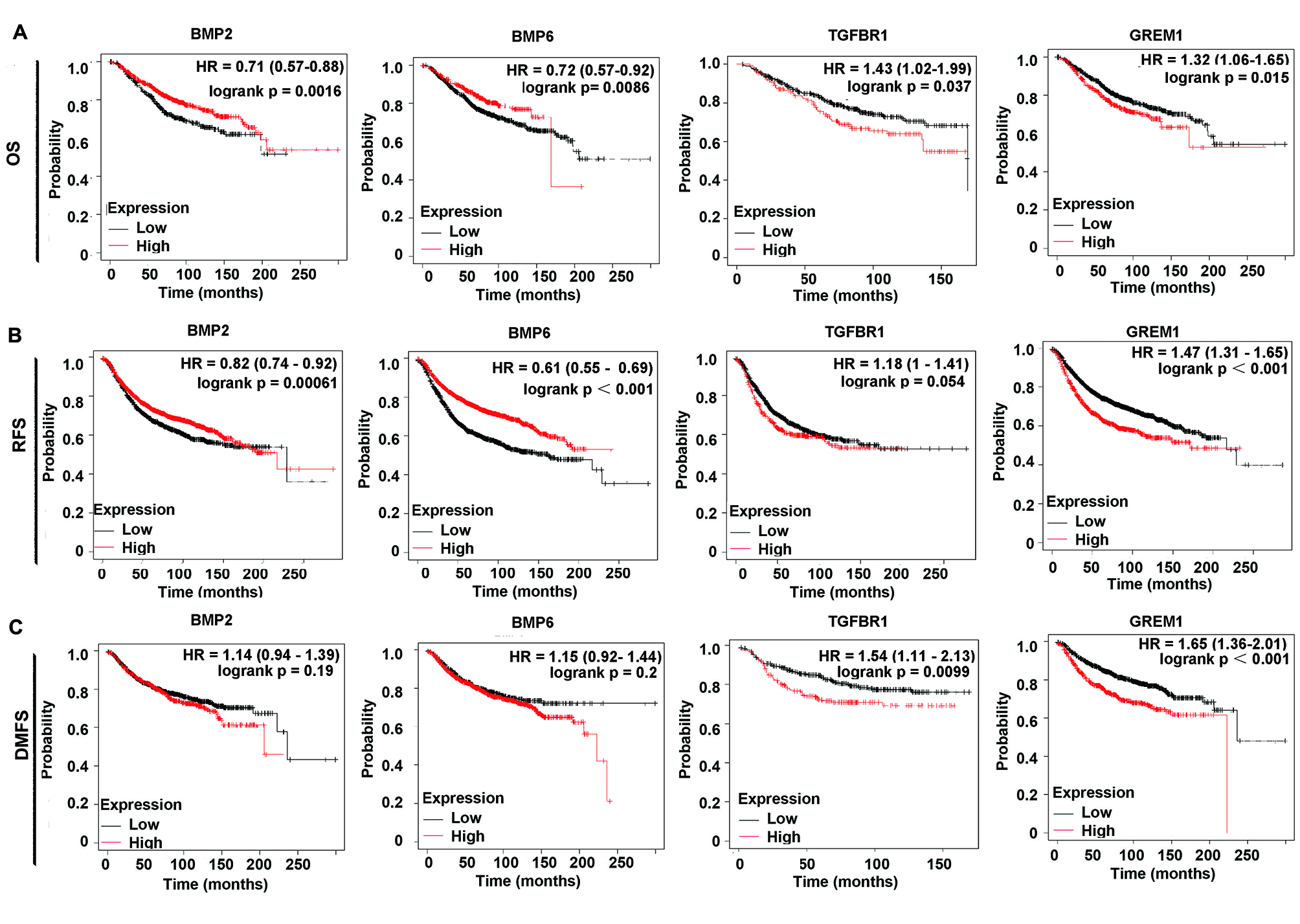

Supplement: Supplementary file 5 [file Image_1.tif]

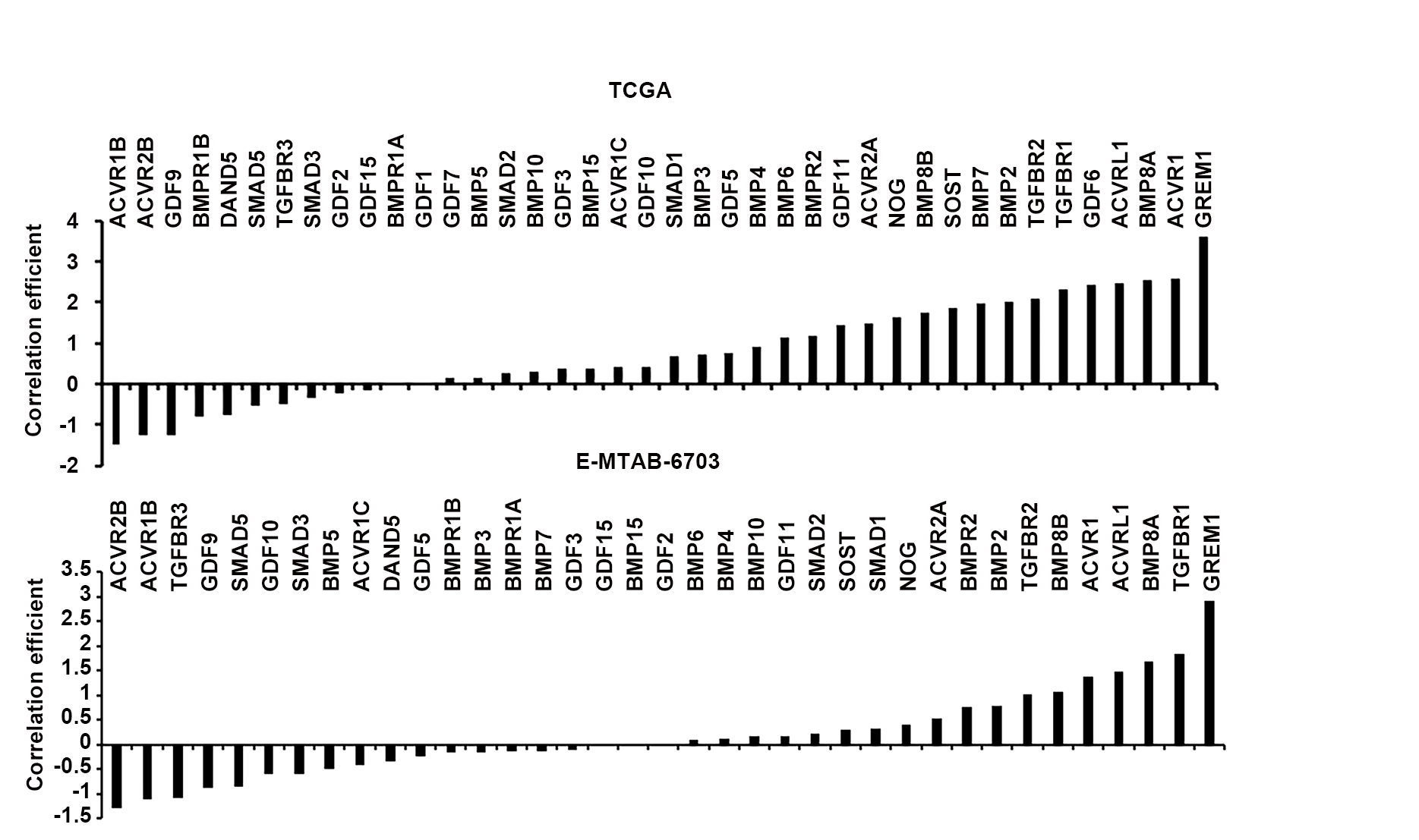

Supplement: Supplementary file 6 [file Image_2.tif]
